# Implant-Supported Rehabilitation Using GBR Combined with Bone Graft on a Reconstructed Maxilla with the Fibula Free Flap

**DOI:** 10.1155/2019/2713542

**Published:** 2019-11-05

**Authors:** S. Di Carlo, V. Valentini, E. Grasso, F. De Angelis, L. Piccoli, A. Quarato, S. Jamshir, E. Brauner

**Affiliations:** Dipartimento Scienze Odontostomatologiche e Maxillo Facciali, Università degli Studi di Roma La Sapienza Facoltà di Medicina e Odontoiatria, Rome, Italy

## Abstract

Alveolar ridge augmentation procedures allow restoring jaw defects due to teeth extractions, periodontal diseases, trauma, or outcomes from a previous surgery. This case report describes a patient suffering from Fibrous Dysplasia of the right upper maxilla surgically reconstructed by fibula free flap. In 2003, four dental implants were placed in the 1.2, 1.3, 1.5, and 1.6 areas. Twelve years later, the onset of peri-implantitis led to the failure of osseointegration with consequent thinning of the fibula flap. To avoid the risk of fracture and to restore the bone volumes necessary for a new implant-prosthetic rehabilitation, we used heterologous biomaterials in combination with a non-reabsorbable membrane, according to the Guided Bone Regeneration (GBR) technique. GBR was performed using the Equimatrix® natural bone mineral matrix, Cytoplast™ Ti-150, a non-reabsorbable titanium-reinforced membrane, and four fastening screws to pin the membrane. After six months, the membrane was removed and two Zimmer® implants 3.7 × 13 mm were placed in the 1.1 and 1.2 areas. A fixed implant-supported prosthesis with a custom-milled titanium bar screwed to the implants was made. Computed tomography (CT) six months after GBR showed a good bone regeneration of 1.5 cm mesiodistal (MD), 1.8 cm buccopalatal (BP), and 2.8 cm in height. The main difficulty of this clinical case concerns the low predictability of success of GBR on a maxillary reconstructed area with a free fibula flap: there is no previous evidence in the literature. Clinical and radiographic exams nowadays show that there is no macroscopic bone reabsorption; however, further research is needed to obtain more information.

## 1. Introduction

Oral implant rehabilitation in patients formerly subjected to partial or total resection of the facial bones is increasingly common [1].

A successful implant rehabilitation requires adequate bone volume which is essential to house the implant and provide esthetics and function [2].

The reconstruction of maxillomandibular defects has undergone considerable progress due to the development of microsurgical techniques. Using vascularized free flaps, nowadays it is possible to reconstruct complex defects providing both soft and bone tissues. The aim of modern surgery is not only the aesthetic outcome of reconstruction but also, and above all, the functional restoration of the stomatognathic apparatus. Aesthetic and functional achievements together enhance the patient's quality of life [3].

The particularity of this case consists in the association of the GBR technique using a bone grafting material and a nonabsorbable membrane on a hemimaxillary reconstruction with the fibula free flap.

## 2. Materials and Methods

A 27-year-old female was referred to the Department of Oral and Maxillofacial Sciences to rehabilitate the upper right quadrant. Her medical history was positive for Fibrous Dysplasia (FD) of the right side of the maxillofacial bones.

Fibrous Dysplasia is a disorder characterized by the replacement of normal bone and marrow with fibrous tissue, resulting in the formation of bone that is weak and prone to expansion. Therefore, most complications result from fracture, deformity, functional impairment, and pain [4].

The patient reported the appearance of a facial swelling at the age of nine.

Due to the increased size of the swelling, in 2001 she underwent right-sided maxillomalar reconstruction for osteodysplastic obliteration of the facial bones.

She was operated at the Maxillofacial Surgery Unit of the hospital Policlinico “Umberto I” in Rome. During surgery, the right half of the maxillary bone was removed, and it was submitted to definitive histological examination, which confirmed the initial diagnosis of Fibrous Dysplasia. A fibula free flap was shaped to reconstruct the alveolar ridge, the orbital margin, the zygomatic knob, and the orbital lateral pillar. The bone, revascularized by Termino-Terminal anastomosis (T-T), was fixed with 6 different plates (2 right-angled plates in the left jaw, 1 X-plate in the frontal process of the jaw, and 3 miniplates in the frontoorbital suture and temporozigomatic suture).

In 2003, the fibula flap was remodelled and 4 dental implants were inserted. The implant abutments were placed during the 12th postoperative month, and the implantation of a resin cement-retained temporary prosthesis was performed. The patient was not finally fit with a permanent prosthesis due to her own negligence.

In 2007, she underwent another surgery for peri-implant inflammatory gingiva removal. After the removal of the inflamed alveolar mucosa at the 1.2-1.5 level, a graft of palatal mucosa was executed. A pink resin for the improvement of the flange prosthetic was, however, added.

**Figure 1:**
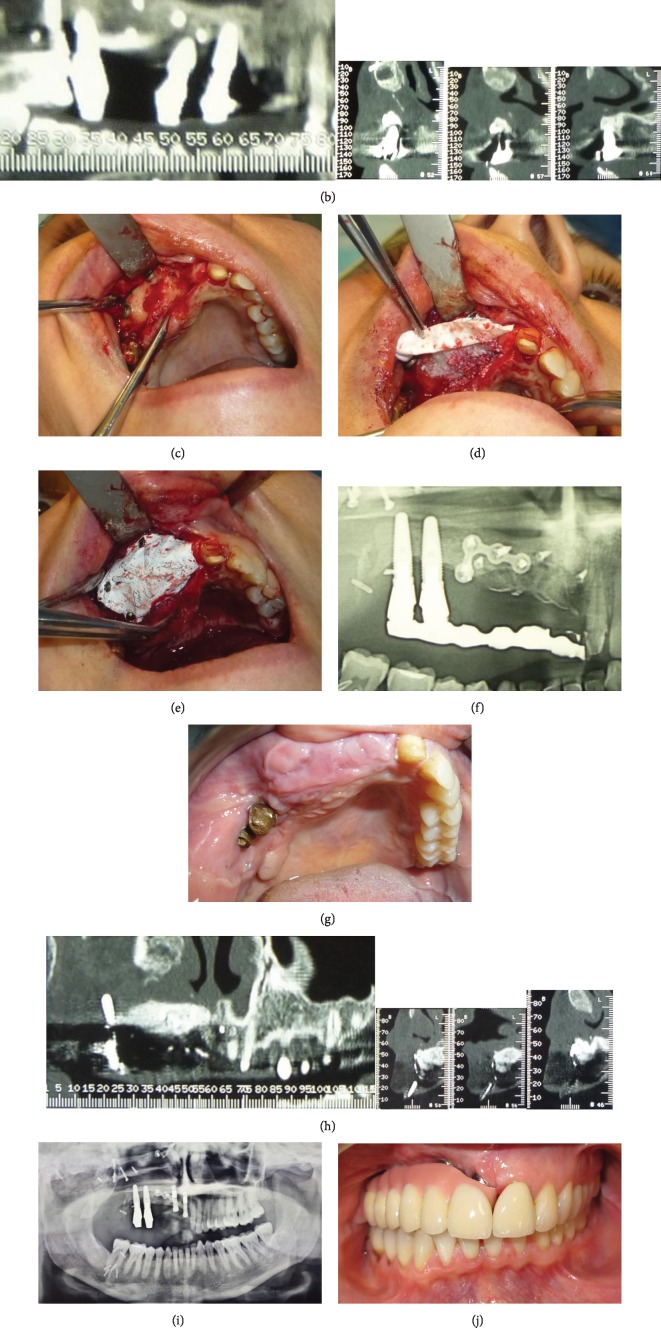
(a) Clinical image shows peri-implantitis, hypertrophy, and severe gingival inflammation. (b) Computerized tomography scan. Preoperative view showing insufficient bone in the apicocoronal and buccolingual dimension to place implants according to the prosthetic needs. (c) Clinical image showing bone defect. (d) Clinical image prior to fixing the membrane on the palatal side showing the graft that completely covers the defect. (e) Clinical image with the membrane completely in place. (f) Panoramic radiograph post-GBR. (g) Clinical image shows tissue healing at three months. (h) Computerized tomography scan. Postoperative view showing robust bone regeneration in the area corresponding to the radiopaque marks on the surgical stent. (i) Panoramic radiograph postimplant surgery. (j) Clinical view of the implant-supported fixed prosthesis. Frontal view. (k) Clinical view of the implant-supported fixed prosthesis. Maxillary occlusal view.

Eight years later, the patient came to our attention. Clinical examination showed the presence of gingival hypertrophy and peri-implantitis (Figure 1(a)). Peri-implantitis had caused the loss of one of the four dental implants, accompanied by a substantial bone resorption.

Our treatment plan was, firstly, to carefully reestablish oral hygiene especially to ablate peri-implant oral mucosa and to allow tissue to heal; therefore, we put the patient into a periodontal maintenance program. The next step was to remove the severely compromised 1.2 implant, carry out a bone graft, and undertake rehabilitation with a tooth-implant-supported fixed partial denture (FPD) including the 2.1 element to ensure better aesthetics and stability of the prosthesis. In preparation for GBR, a temporary prosthesis which did not lean on the graft to avoid inevitable bone reabsorption was performed.

After orthopanthomography (OPT) and computer tomography (CT) X-ray analysis, and considering the amount of bone resorption, we opted for the GBR technique. The bone defect was calculated on the basis of three-dimensional (3D) computational analysis of the CT imaging and measured 1.9 cm MD, 1.8 cm BP, and 2.8 cm in height (Figure 1(b)).

The patient received a prophylactic preoperative dose of an oral antibiotic (2 g amoxicillin/clavulanic acid 1 hour before surgery), and a mouth rinse with 15 mL of 0.2% chlorhexidine solution was used before surgery for 1 minute. After administration of local anesthesia, induced with mepivacaine 2% + epinephrine 1 : 100,000 (2% carbocaine) to ensure deeper pain control and contain bleeding, the compromised implant 1.2 was removed. Then, a ridge incision was performed, and bone tissue was exposed (Figure 1(c)). Multiple cortical perforations, which created openings for osteopromotion, were then made to stimulate blood and cell migration from the bone marrow spaces to the regeneration area. A nonresorbable titanium-reinforced membrane (Cytoplast Ti-150) was positioned to create a barrier effect against epithelium cells and to maintain the grafted space. The membrane was pinned by three buccal osteosynthesis screws (distal, central, and mesial); then, a graft material was positioned in the defective area. We used Equimatrix (0.2-1.0 mm) natural bone mineral matrix, an equine-derived natural bone graft substitute with a complex, porous network that closely resembles natural human bone. After positioning the graft material which was left protruding from the crest, another palatal (distal) screw was used for fixing the membrane (Figures 1(c)–1(e)). A tension-free suture was performed to allow healing by first intention and to prevent membrane exposure, a complication that may occur in bone regeneration.

## 3. Results

After a healing period of 6 months, newly formed bone beneath the membrane was obtained (Figures 1(f) and 1(g)). Another surgical procedure was required to remove the nonresorbable membrane with the osteosynthesis screws and to place the implants. A CT Dentascan with a radiological template inserted in the oral cavity was performed. Computed tomography (CT) six months after GBR showed a bone regeneration of 1.5 cm mesiodistal, 1.8 cm buccopalatal, and 2.8 cm in height (Figure 1(h)). With the aid of this procedure, it was decided to place two Zimmer Trabecular Metal™ implants (Ø 3.7 mm, L 13.0 mm) in the maxillary canine and central incisive area. The clinical situation after removal of the membrane showed a compact and non-reabsorbed graft. The implant insertion torque was greater than 32 Newtons, and implant stability had a value greater than 60 ISQ. Measurements were made by the Osstell® system. They confirmed good bone quality and a good bone-implant interface. Primary intention healing had been obtained: periosteal incisions were performed to make the flap passive and it was sutured using Donati stiches with an absorbable suture to close the flap and simple sutures with a nonabsorbable suture on the occlusal margin. After the procedure, the patient was told to continue the antibiotic therapy for 6 days (1 g every 8 h), to use a 0.2% chlorhexidine mouthwash 3 times a day for two weeks, and to take an anti-inflammatory drug as needed. After 6 months during which the membrane had not been exposed, implant uncovering was performed, and healing screws were inserted (Figure 1(i)).

After 3 months, the soft tissue had healed completely and prosthetic rehabilitation for implant-supported fixed dentures was begun. The primary structure is a custom-milled titanium bar screwed onto the dental implants. The secondary structure has a series of geometrical elements shaped inversely to the grooves of the primary structure and consists of a metal covered with porcelain for the teeth and composite for the pink gum (Figures 1(j) and 1(k)).

The occlusal plane presented alteration. In fact, since the patient had been affected by Fibrous Dysplasia during developmental age, mesiodistal lower plane growth was present. Therefore, onlays made using an indirect method were inserted on the 4.4, 4.5, 4.6, and 4.7 elements to restore the occlusal plane.

## 4. Discussion

Facial reconstructive surgery has seen great progress over the last century, improving the quality of life for patients following ablative surgery, by restoring both function and aesthetic outcome. Microvascular flap employment and the development of implantology have largely helped to achieve these goals [5–7].

With regard to the flaps employed, the fibula is one of the most versatile options. The use of the fibula microvascular flap for maxillomandibular reconstruction and especially for dental rehabilitation has the following major advantages: its length makes it possible to use up to 25 cm of bone for the reconstruction and it provides the chance of shaping it; its extraordinary periosteal vascularization gives the possibility of performing multiple osteotomies; and it has lower morbidity in the donor area [3, 8].

The bone structure of the fibula is very similar to the mandible, where cortical bone dominates. This structural similarity allows a good osteintegration of dental implants, the same as for the mandible [9–11]. Therefore, in this clinical case the problem was not only to achieve regeneration and to insert implants but also to consider the type of autologous bone already grafted. Surgery regarded the upper jaw but was the same as that for the mandible. Furthermore, the absence of baseline bone implicated a risk a pathological fracture in that area. Then, we had to pay close attention to the risk of fractures.

Various methods have been developed to increase bone volume and augment new tissue growth [12–15]: (1) distraction osteogenesis, which describes the surgical induction of a fracture and the subsequent gradual separation of the two bone ends to create spontaneous bone regeneration between the two fragments; (2) osteoinduction, which employs appropriate growth factors and/or stem/osteoprogenitor cells to encourage new bone formation; (3) osteoconduction, in which a grafting material serves as a scaffold for new bone formation; and (4) guided bone regeneration (GBR), which provides spaces using barrier membranes that subsequently become filled with new bone [16, 17].

Among these different regenerative techniques, GBR seemed to us the best option because it was necessary to fill a predominantly vertical bone defect [18, 19]; therefore, the stiffness of the nonresorbable titanium membrane was used to support the soft tissues and thus allow the formation of new bone [20, 21]. To ensure successful GBR, four principles need to be met: exclusion of epithelium and connective tissue, space maintenance, stability of the fibrin clot, and primary wound closure.

This case goes to show that by following the operative protocol scrupulously and respecting its principles, it is possible to obtain good bone regeneration even in complex cases [22].

The main difficulty of the present clinical case concerned the low predictability of success of GBR on a maxillary reconstructed area with a free fibula flap. There is no evidence to support the claims made in the literature. To maximize success in similar advanced clinical cases, a scrupulous surgical protocol must be followed. Furthermore, it is important for the dentist to approach these patients knowing the kind of surgery they received because this aspect will influence rehabilitative choices [23–26]. The amount of regeneration we achieved was 1.5 cm MD, 1.8 cm BP, and 2.8 cm in height. Follow-up controls of patients must be performed every 3-4 months for monitoring and reevaluation. Clinical and radiographic findings (bone sounding and intraoral X-ray images) suggested that there was no significant bone resorption. The patient was satisfied with the prosthesis both esthetically and functionally and reported significant improvements in oral function and psychosocial activities. However, further research in this area and long-term monitoring are needed to obtain more information.
